# Changes of [^18^F]FDG-PET/CT quantitative parameters in tumor lesions by the Bayesian penalized-likelihood PET reconstruction algorithm and its influencing factors

**DOI:** 10.1186/s12880-021-00664-7

**Published:** 2021-09-16

**Authors:** Yao Liu, Mei-jia Gao, Jie Zhou, Fan Du, Liang Chen, Zhong-ke Huang, Ji-bo Hu, Cen Lou

**Affiliations:** Department of Nuclear Medicine, Sir Run Run Shaw Hospital, Zhejiang University School of Medicine, 3 East Qingchun Rd, Jianggan District, Hangzhou, 310000 Zhejiang People’s Republic of China

**Keywords:** Cancer, PET/CT, BPL, SUV

## Abstract

**Background:**

To compare the changes in quantitative parameters and the size and degree of ^*1*8^F*-*fluorodeoxyglucose ([^18^F]FDG) uptake of malignant tumor lesions between Bayesian penalized-likelihood (BPL) and non-BPL reconstruction algorithms.

**Methods:**

Positron emission tomography/computed tomography images of 86 malignant tumor lesions were reconstructed using the algorithms of ordered subset expectation maximization (OSEM), OSEM + time of flight (TOF), OSEM + TOF + point spread function (PSF), and BPL. [^18^F]FDG parameters of maximum standardized uptake value (SUVmax), SUVmean, metabolic tumor volume (MTV), total lesion glycolysis (TLG), and signal-to-background ratio (SBR) of these lesions were measured. Quantitative parameters between the different reconstruction algorithms were compared, and correlations between parameter variation and lesion size or the degree of [^18^F]FDG uptake were analyzed.

**Results:**

After BPL reconstruction, SUVmax, SUVmean, and SBR were significantly increased, MTV was significantly decreased. The difference values of %ΔSUVmax, %ΔSUVmean, %ΔSBR, and the absolute value of %ΔMTV between BPL and OSEM + TOF were 40.00%, 38.50%, 33.60%, and 33.20%, respectively, which were significantly higher than those between BPL and OSEM + TOF + PSF. Similar results were observed in the comparison of OSEM and OSEM + TOF + PSF with BPL. The %ΔSUVmax, %ΔSUVmean, and %ΔSBR were all significantly negatively correlated with the size and degree of [^18^F]FDG uptake in the lesions, whereas significant positive correlations were observed for %ΔMTV and %ΔTLG.

**Conclusion:**

The BPL reconstruction algorithm significantly increased SUVmax, SUVmean, and SBR and decreased MTV of tumor lesions, especially in small or relatively hypometabolic lesions.

## Background

^18^F-fluorodeoxyglucose positron emission tomography combined with computed tomography ([^18^F]FDG-PET/CT) imaging technology is widely used in clinical practice, not only for disease diagnosis especially of malignant tumors but also for treatment monitoring. The quantitative analysis of a lesion’s characteristics is conducive to improving disease diagnosis and treatment monitoring [1]. However, the accurate quantification of PET/CT parameters is challenged by lesion size, volume, and contrast, which are mainly influenced by reconstruction algorithm [2]. As an emerging reconstruction technology, the Bayesian penalized-likelihood (BPL) reconstruction algorithm takes the accuracy of PET/CT quantitative parameters a step forward without sacrificing high-quality images [3, 4]. Different from the traditional ordered subset expectation maximization (OSEM) reconstruction algorithm, BPL effectively converges with PET imaging and improves image quality by controlling the noise and potentially increasing the number of iterations in high-activity lesions, thereby achieving the best balance between image quality and accurate quantification.

Previous studies have shown that changes of [^18^F]FDG uptake in tumor lesions by BPL construction can be affected by lesion size and the degree of [^18^F]FDG uptake [4–8]. These studies showed that BPL can increase [^18^F]FDG uptake in non-small cell lung cancer lesions, especially small lesions [6–8], resulting in improvements of the sensitivity and accuracy for lung nodule diagnosis. Furthermore, changes in maximum standardized uptake value (SUVmax) in lesions reconstructed by BPL, which have different levels of [^18^F]FDG, are also significantly different [6].

In the past, research mainly focused on certain kinds of malignant diseases, such as lung cancer, liver cancer, prostate cancer and their metastatic diseases. However, there were many other kinds of malignant tumors that were less studied in clinical practice. The role of BPL reconstruction algorithm on different kinds of malignant tumors remains unsolved. Hence, we conducted this study to analyze the effect of BPL reconstruction technology on [^18^F]FDG standard uptake parameters and volume metabolism parameters of different kinds of malignant tumor lesions. Additionally, the relationship between these parameters and the size or degree of FDG uptake in lesions was also explored. We anticipated to find an accurate reconstruction algorithm that may provide clear visibility of small malignant lesions, thus improving tumor staging, treatment planning, response monitoring, and prognosis prediction.

## Methods

### Patients

We retrospectively searched our database for patients who underwent [^18^F]FDG PET/CT examination in our hospital’s nuclear medicine department from May 2019 to October 2019 due to suspicious or confirmed malignant tumors. Patients with hypermetabolic lesions on a PET scan, which were confirmed to be primary malignant tumors or metastatic lesions by histopathology, were eligible for this study. The study was approved by the institutional review board of Sir Run Run Shaw Hospital (Zhejiang, China). All procedures performed in studies involving human participants were in accordance with the ethical standards of the institutional and/or national research committee and with the 1964 Helsinki declaration and its later amendments or comparable ethical standards. The informed consent requirement for this retrospective study was waived by The Ethics Committee of Sir Run Run Shaw Hospital, Affiliated to School of Medicine, Zhejiang University.

### [^18^F]FDG-PET/CT imaging protocol

All patients underwent a PET/CT scan with a novel digital detector scanner (Discovery MI; GE Healthcare, Milwaukee, WI, USA) based on PET/CT procedure guidelines for imaging [9]. The patients were required to fast for at least 6 h before PET/CT scans, and the serum glucose levels were required to be below 200 mg/dL at the time of [^18^F]FDG injection. Then the patients were given an intravenous injection of 3.7 MBq/kg FDG. Imaging commenced approximately 60 min after the injection and covered the skull base to upper thighs with the patients’ arms over their heads. Helical CT scan was performed with a pitch of 0.984, voltage of 120 kV, auto mA ranging from 30 to 180 mA, and noise index of 25. And the CT scans for attenuation correction were conducted without contrast agent. After the CT scan, the PET scan was immediately acquired in a three-dimensional mode with anatomical region identical to the CT scan. The PET acquisition was 2.79 mm in slice thickness and 1.5 min per bed position, with 6–8 bed positions per patient (depending on patient size) and an overlap of 23% (17 slices).

### PET reconstruction

PET images were reconstructed using OSEM (without point spread function modeling; GE Healthcare) with two iterations, 24 subsets, and a 6.4 mm Gaussian filter, which is a typical standard of care. After PET acquisition, the raw data were reconstructed using four different reconstruction algorithms per patient: 1) OSEM (two iterations/24 subsets, 6.4 mm Gaussian filter); 2) OSEM (two iterations/24 subsets, 6.4 mm Gaussian filter) + time of flight (TOF); 3) OSEM (two iterations/24 subsets, 6.4 mm Gaussian filter) + TOF + PSF; and 4) BPL with a β-value of 400. The β-value is a penalization factor associated with the regularization effect on BPL reconstruction [10]. Among them, reconstruction algorithms 1–3 were non-BPL algorithms, and 4 was a BPL algorithm. All datasets were reconstructed with a 256 × 256-pixel matrix (voxels of 2.73 × 2.73 × 2.78 mm^3^). The standard PET reconstruction algorithm used at our center is BPL, namely the Q.Clear reconstruction algorithm (GE Healthcare). The reconstructed PET images were viewed on the GE Advantage Workstation (AW4.7; GE Healthcare), and the CT components were available for image fusion. Four reconstructed PET/CT images were selected to show the same slice of a lesion, and volume of interests (VOIs) of lesions were automatically outlined. To compute the metabolic total volume (MTV), a 42% VOI on the SUVmax was selected for each lesion in all reconstructions. TLG was calculated as MTV multiplied by SUVmean. Quantitative parameters including standard uptake parameters (SUVmax and SUVmean), and volume metabolism parameters (MTV and total lesion glycolysis [TLG]) of the lesion VOIs were automatically measured. Meanwhile, a 1 cm^3^ spherical VOI was drawn on the parenchyma of the right lobe of liver, and the SUVmean of the liver VOI was measured as a quantitative background parameter. The tumor apparent signal-to-background ratio (SBR) was defined as the lesion’s SUVmax divided by the liver SUVmean and by calculating the change rate of the quantitative parameter (%Δ) between the BPL group and non-BPL group, such as %ΔSUV = (SUV_BPL_-SUV _non-BPL_) × 100%/SUV_non-BPL_.

### Statistical analysis

All data were non-normally distributed and are presented as the median and inter quartile range (IQR) (P25, P75). The Kruskal–Wallis H test with Bonferroni correction for the post hoc test was used to compare the differences in quantitative parameters between reconstruction groups. The correlation between the change rate of quantitative parameters and the size and degree of the lesion’s FDG uptake was determined by Spearman’s correlation analysis. The results were considered statistically significant at *P* < 0.05. All statistical analyses were performed using IBM SPSS Statistics 22.0 (IBM Corporation, Armonk, NY, USA).

## Results

### Clinicopathological characteristics

A total of 86 lesions were obtained from 53 patients (30 males and 23 females) who met the criterion. The median and IQR (P25, P75) of long-axis diameter of the lesions were 18.5 and 18.3 mm (3.2, 91.5). The median and IQR (P25, P75) of age were 63 and 16.5 years (23, 87). The median and IQR (P25, P75) of weight were 58.1 and 9.25 kg (41, 85.8). The malignant tumors originated from the head, neck, lung, digestive tract, lymphatic system, bone, or urinary system (Table 1).

**Table 1:** Clinicopathological characteristics

Variable	Number (%)/Median, IQR (P25, P75), range
Female/male, n (%)	30 (56.60%)/23 (43.40%)
Age, years	63.00, 16.50 (53.00, 69.50), 24.00–89.00
Body weight, kg	58.10, 9.25 (53.87, 63.12), 41.00–85.80
BMI (kg/m^2^)	21.75, 4.38 (19.53, 23.90), 18.25–30.25
PET/CT scan post-injection time, min	56.00, 17.50 (50.5, 68.00), 45.00–88.00
Blood glucose level at time of injection, mg/dl	113.50, 23.96 (104.04, 127.91), 73.80–185.40
Tumor length, mm	18.50, 18.30 (11.70, 30.00), 3.20–91.50
*The source of malignant tumors*	
Head and neck	14 (23.33%)
Lung	9 (15.00%)
Gastrointestinal system	13 (21.67%)
Lymphatic system	9 (15.00%)
Bone	4 (6.67%)
Urinary system	11 (18.33%)

### Influence of different reconstruction algorithms on the quantitative parameters of PET-CT

[^18^F]FDG standard uptake parameters (SUVmax, SUVmean, and SBR) of the tumor lesions are shown in Table 2. The SUVmax, SUVmean, and SBR values for the PET scans reconstructed by the BPL algorithm were significantly greater than those by OSEM, OSEM + TOF, or OSEM + TOF + PSF algorithm (X^2^ = 38.78, X^2^ = 38.21, X^2^ = 33.62, respectively; all *P* < 0.001). After pair-wise comparison, the SUVmax, SUVmean, and SBR values were significantly different between OSEM and BPL, OSEM + TOF and BPL, and OSEM + TOF + PSF and BPL (all adjusted *P* < 0.001 for the SUVmax; all adjusted *P* < 0.001 for the SUVmean; adjusted *P* = 0.001, 0.001, 0.008 for the SBR). However, the SUVmax, SUVmean and SBR values had no significantly differences between OSEM and OSEM + TOF (adjusted *P* = 1.000, 0.283, and 1.000, respectively), OSEM and OSEM + TOF + PSF (adjusted *P* = 1.000, 0.305, and 1.000, respectively), and OSEM + TOF and OSEM + TOF + PSF (adjusted *P* = 1.000, 0.144, and 1.000, respectively). In contrast, the MTV for the PET scans reconstructed by the BPL algorithm was significantly lower than that by OSEM, OSEM + TOF, or OSEM + TOF + PSF algorithm (X^2^ = 12.69, *P* = 0.005). After pair-wise comparison, the MTVs were different between BPL and OSEM, and between BPL and OSEM + TOF (adjusted *P* = 0.006, 0.028, respectively). The MTVs were not significantly different between BPL and OSEM + TOF + PSF, OSEM + TOF + PSF and OSEM + TOF, OSEM + TOF + PSF and OSEM, and OSEM + TOF and OSEM (adjusted *P* = 0.343, 1.000, 1.000 and 1.000, respectively). Regarding the TLG, no significant difference was found among the four reconstruction algorithms (X^2^ = 0.72, *P* = 0.868). Regarding the TLG, no significant difference was found among the four reconstruction algorithms (X^2^ = 0.72, *P* = 0.868).

**Table 2:** Comparison of quantitative parameters of [^18^F]FDG using different reconstruction algorithms for malignant tumor lesions (Kruskal–Wallis H test)

Reconstruction algorithms	Quantitative parameters (Median P25, P75)				
	SUVmax	SUVmean	SBR	MTV	TLG
OSEM	8.07	4.75	4.36	5.15	23.86
	(5.37, 11.39)	(3.04, 7.18)	(2.79, 6.43)	(3.03, 12.48)	(11.17, 70.64)
OSEM + TOF	8.38	5.01	4.65	4.86	25.97
	(6.08, 12.08)	(3.51, 7.53)	(3.23, 6.76)	(2.57, 12.54)	(11.35, 74.00)
OSEM + TOF + PSF	9.01	5.39	5.2	4.31	23.62
	(6.85, 12.67)	(3.97, 7.73)	(3.74, 7.31)	(2.21, 11.05)	(10.06, 73.92)
BPL	11.75	7.42	6.68	3.08	22.59
	(9.97, 16.82)	(5.83, 10.56)	(5.19, 8.94)	(1.13, 9.66)	(7.02, 79.95)
X2	38.78	38.21	33.62	12.26	0.72
p value	< 0.001	< 0.001	< 0.001	0.005	0.868

The different rates of quantitative parameters such as %ΔSUVmax, %ΔSUVmean, %ΔSBR, and the absolute value of %ΔMTV between OSEM and BPL were 42.2%, 46.7%, 42.8%, and 37.6%, respectively, which were significantly higher than those between OSEM + TOF and BPL (40.0%, 38.5%, 33.6%, and 33.2%, respectively) and between OSEM + TOF + PSF and BPL (26.7%, 27.9%, 21.4%, and 21.7%, respectively) (X^2^ = 25.45 and *P* < 0.001 for %ΔSUVmax, X^2^ = 22.37 and *P* < 0.001 for %ΔSUVmean, X^2^ = 35.26 and *P* < 0.001 for %ΔSBR, and X^2^ = 8.67 and *P* = 0.013 for %ΔMTV). After pair-wise comparisons, the %ΔSUVmax, %ΔSUVmean, %ΔSBR, and %ΔMTV of OSEM + TOF + PSF vs. OSEM + TOF were significantly different from those of BPL (adjusted *P* = 0.008, adjusted *P* < 0.001, adjusted *P* = 0.003, adjusted *P* = 0.046, respectively); the %ΔSUVmax, %ΔSUVmean, %ΔSBR, and %ΔMTV of OSEM + TOF + PSF vs. OSEM were significantly different from those of BPL (adjusted *P* = 0.022, adjusted *P* < 0.001, adjusted *P* < 0.001, adjusted *P* = 0.024, respectively). However, compared to BPL, the %ΔSUVmax, %ΔSUVmean, %ΔMTV, and %ΔSBR had no significant differences between OSEM + TOF and OSEM (adjusted *P* = 0.139, 0.124, 1.000, 0.025, respectively). There was no significant difference in %ΔTLG between the reconstruction algorithms (X^2^ = 2.88, *P* = 0.238; Table 3).

**Table 3:** Comparison of the change rate of quantitative parameters in OSEM, OSEM + TOF, and OSEM + TOF + PSF groups as compared to BPL group using Kruskal–Wallis H test

Reconstruction algorithms	Change rate of quantitative parameters % (median P25, P75)				
	%ΔSUVmax	%ΔSUVmean	%ΔSBR	%ΔMTV	%ΔTLG
OSEM	42.20%	46.70%	42.80%	− 37.60%	− 2.60%
	(26.1%, 85.1%)	(27.0%, 90.4%)	(27.1%, 85.7%)	(58.8%, − 14.0%)	(− 23.1%, 9.2%)
OSEM + TOF	40.00%	38.50%	33.60%	− 33.20%	− 6.80%
	(20.9%, 68.1%)	(21.3%, 71.5%)	(19.2%, 64.6%)	(55.6%, 17.5%)	(− 22.9%, 3.8%)
OSEM + TOF + PSF	26.70%	27.90%	21.40%	− 21.70%	− 3.30%
	(15.4%, 49.0%)	(15.2%, 54.9%)	(12.5%, 46.1%)	(− 42.8%, − 8.3%)	(− 15.0%, 7.5%)
X2	25.45	22.37	35.32	8.67	2.87
P value	< 0.001	< 0.001	< 0.001	0.013	0.238

### Relationship between the change rate of quantitative parameters and the size or degree of FDG uptake in the lesions

**Fig. 1:**
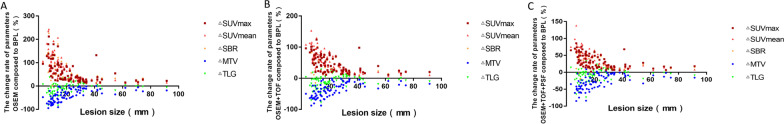
Scatter plot of correlation analysis between the change rate of quantitative parameters and the lesion size in the OSEM (**a**), OSEM + TOF (**b**), or OSEM + TOF + PSF (**c**) group compared with the BPL group. The change rate of quantitative parameters increased for ΔSUVmax, ΔSUVmean, and ΔSBR, and decreased for ΔMTV and ΔTLG, as the lesion size decreased

**Fig. 2:**
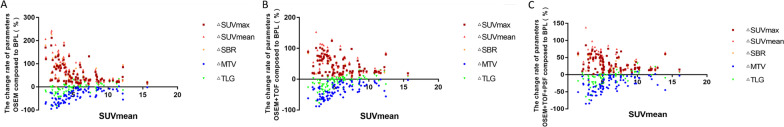
Scatter plot of correlation analysis between the degree of lesion uptake SUVmean and the change rate of quantitative parameters in the OSEM (**a**), OSEM + TOF (**b**), or OSEM + TOF + PSF (**c**) group compared with the BPL group. The lower amount of lesion uptake resulted in increased change rates for ΔSUVmax, ΔSUVmean, and ΔSBR, and decreased change rates for ΔMTV and ΔTLG

**Fig. 3:**
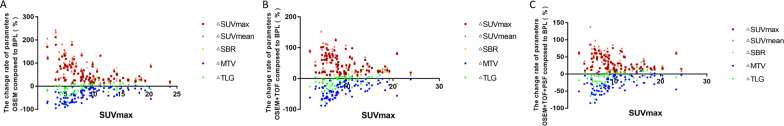
Scatter plot of correlation analysis between SUVmax and the change rate of quantitative parameters in the OSEM (**a**), OSEM + TOF (**b**), or OSEM + TOF + PSF (**c**) group compared with the BPL group. The lower amount of lesion uptake resulted in increased change rates for ΔSUVmax, ΔSUVmean, and ΔSBR, and decreased change rates for ΔMTV and ΔTLG

The %ΔSUVmax, %ΔSUVmean, and %ΔSBR in the OSEM, OSEM + TOF, and OSEM + TOF + PSF reconstruction groups were negatively correlated with the size (*P* < 0.001) and degree of FDG uptake (*P* < 0.05) of the lesions, whereas the %ΔMTV and %ΔTLG were positively correlated with the size (*P* < 0.001) and degree of FDG uptake (*P* < 0.001) of the lesions (Tables 4, 5, 6; Figs. 1, 2, 3).

**Table 4:** Correlation between the change rate of quantitative parameters and the size of lesions in the OSEM, OSEM + TOF, and OSEM + TOF + PSF groups as compared to the BPL group using Spearman’s correlation analysis

Reconstruction algorithms	Change rate of quantitative parameters P value (r)				
	%ΔSUVmax	%ΔSUVmean	%ΔSBR	%ΔMTV	%ΔTLG
OSEM	< 0.001 (− 0.786)	< 0.001 (− 0.867)	< 0.001 (− 0.708)	< 0.001 (0.716)	< 0.001 (0.454)
OSEM + TOF	< 0.001 (− 0.714)	< 0.001 (− 0.817)	< 0.001 (− 0.581)	< 0.001 (0.699)	< 0.001 (0.399)
OSEM + TOF + PSF	< 0.001 (− 0.709)	< 0.001 (− 0.822)	< 0.001 (− 0.570)	< 0.001 (0.648)	< 0.001 (0.343)

**Table 5:** Correlation between the change rate of quantitative parameters and the SUVmean of lesions in the OSEM, OSEM + TOF, and OSEM + TOF + PSF groups as compared to the BPL group using Spearman’s correlation analysis

Reconstruction algorithms	Change rate of quantitative parameters P value (r)				
	%ΔSUVmax	%ΔSUVmean	%ΔSBR	%ΔMTV	%ΔTLG
OSEM	< 0.001 (− 0.572)	< 0.001 (− 0.627)	< 0.001 (− 0.524)	< 0.001 (0.730)	< 0.001 (0.668)
OSEM + TOF	< 0.001 (− 0.368)	< 0.001 (− 0.425)	0.0139 (− 0.274)	< 0.001 (0.661)	< 0.001 (0.663)
OSEM + TOF + PSF	< 0.001 (− 0.387)	< 0.001 (− 0.420)	0.0138 (− 0.265)	< 0.001 − 0.628	< 0.001 (0.658)

**Table 6:** Correlation between the change rate of quantitative parameters and the SUVmax of lesions in the OSEM, OSEM + TOF, and OSEM + TOF + PSF groups compared to the BPL group using Spearman’s correlation analysis

Reconstruction algorithms	Change rate of quantitative parameters p value (r)				
	%ΔSUVmax	%ΔSUVmean	%ΔSBR	%ΔMTV	%ΔTLG
OSEM	< 0.001	< 0.001	< 0.001	< 0.001	< 0.001
	(− 0.563)	(− 0.610)	(− 0.516)	− 0.702	− 0.635
OSEM + TOF	< 0.001	< 0.001	0.0139	< 0.001	< 0.001
	(− 0.367)	(− 0.425)	(− 0.272)	− 0.643	− 0.623
OSEM + TOF + PSF	< 0.001	< 0.001	0.0138	< 0.001	< 0.001
	(− 0.372)	(− 0.405)	(− 0.253)	− 0.604	− 0.64

## Discussion

Our study demonstrated that the BPL reconstruction algorithm significantly increased the SUVmax, SUVmean, and SBR while significantly decreasing the MTV of malignant tumor lesions compared with the OSEM and OSEM + TOF reconstruction algorithms. The changes were more obvious in small tumor lesions or lesions with relatively hypometabolic tumors.

[^18^F]FDG PET/CT is widely used in clinical practice for the quantitative analysis of malignant lesions such as diagnosis, treatment monitoring, and prognosis assessment. Signal acquisition and reconstruction algorithms have the greatest impact on the accuracy of quantitative parameters. Currently, the OSEM algorithm using repeated iterations remains the primary method used for the reconstruction of PET/CT imaging. With increasing iterations, focal maxima of activity further increases, and may overestimate the true activity, as well as image noise increases. To limit the image noise, OSEM data are usually smoothed with a filter. However, this decreases contrast recovery of “hot” lesions which may result in underestimation of the true activity. Furthermore, OSEM algorithm may not be fully convergent. Based on this context, combining or replacing OSEM with other reconstruction algorithms may improve the accuracy of quantitative parameters of tumor lesions’ [^18^F]FDG uptake. BPL is a reconstruction method that can achieve more appropriate convergence through more iterations, and control the image noise at a suitable level [3]. The noise suppression function can be controlled by adjusting the penalization factor (β), which is the only variable that allows user to input. Reducing the penalization factor (β) increases the contrast recovery coefficients and the background variability. In a NEMA phantom research by Teoh et al., a higher contrast recovery coefficient was achieved by BPL reconstruction than those by OSEM + TOF and OSEM + PSF; that is, the accuracy of the quantitative parameters was improved by the BPL algorithm [1, 10, 11]. And, the signal-to-noise ratio can be improved by BPL at equal contrast recovery [12]. These advantages of BPL reconstruction can increase the accuracy of [^18^F]FDG quantitative parameters without compromising the image quality compared with the OSEM method, with a particular improvement in small tumor lesions.

Our study compared the [^18^F]FDG standard uptake parameters and volume metabolism parameters of malignant tumor lesions in four PET/CT reconstruction algorithms. The results showed that the BPL reconstruction algorithm, which based on TOF and PSF, significantly increased the standard uptake parameters (SUVmax, SUVmean, and SBR) of [^18^F]FDG in tumors, while MTV was significantly decreased. And, these standard uptake parameters were significantly higher in BPL than in OSEM, OSEM + TOF, and OSEM + TOF + PSF by pair-wise comparisons. But, MTV in BPL was significantly lower than OSEM and OSEM + TOF. Murphy et al. compared the effects of BPL and OSEM reconstruction algorithms on [^18^F]FDG metabolic parameters in malignant solitary pulmonary nodules in the context of non-small cell lung cancer, mediastinal metastatic lymph nodes, and colorectal cancer with liver metastases [6, 7, 13]. Their results showed that the SUVmax of corresponding lesions reconstructed by the BPL were 8.3 (3.2–13.4), 7.0 (1.3–25.3), and 11.6 (2.6–25.7), respectively, which were significantly higher than those by OSEM (*P* < 0.001). Similarly, Reynés-Llompart et al*.* compared the [^18^F]FDG metabolic parameters SUVmax and SUVmean of tumor lesions between PSF and BPL reconstruction algorithms. Their results were consistent with our findings, but no significant difference in mean lesion volume was found between these two algorithms. This may be for the reason that the convergence function was affected by different size lesions when using BPL algorithm [11]; the research objects were lung nodules in the study by Reynés-Llompart’s et al., whereas the range of lesion size was wide in our study. Matti et al. noted that the BPL reconstruction algorithm can improve the accuracy of SUVmax and SUVmean of [^18^F]FDG in hypermetabolized lesions without changing the metabolic parameters of background tissue, thus resulting in the improvement of SBR of tumor lesions and image quality [12]. However, some literature has suggested that BPL only increases the apparent [^18^F]FDG metabolic parameters and may not improve the ability to distinguish benign and malignant lesions [6, 7]. When lesions had low SUV, [^18^F]FDG PET/CT often could not accurately evaluate true metabolic activity, and when both non-malignant and malignant lesions had a high degree of [^18^F]FDG uptake, [^18^F]FDG PET/CT was not specific for distinguishing the nature of nodular lesions. Thus, the benefit of using BPL is mainly to provide better lesion visibility and more accurate quantitative parameters for clinical practices.

In this study, we found that the different rates regarding %ΔSUVmax, %ΔSUVmean, %ΔSBR, and the absolute values of %ΔMTV between the BPL and OSEM + TOF were significantly higher than those between the BPL and OSEM + TOF + PSF. Similar results were observed between OSEM and OSEM + TOF + PSF as compared to BPL. Furthermore, %ΔSUVmax, %ΔSUVmean, and %ΔSBR in the OSEM, OSEM + TOF, and OSEM + TOF + PSF groups were significantly negatively correlated with lesion size and the degree of lesion FDG uptake, suggesting that the BPL reconstruction technology had a more significant convergent effect on small lesions and low degree [^18^F]FDG uptake lesions. Consequently, by increasing metabolic parameters and the detection sensitivity of small lesions, BPL may help find out the small lesions with low degree [^18^F]FDG uptake and provide further guidance for tumor staging, evaluation, and treatment decisions in clinical practice.

Consistent with a study by Parvizi [5], our results also confirmed that BPL reduced the MTV of tumor lesions. There are two possible reasons, one of which is the effective convergence by BPL that PET images with lower noise and higher contrast through image noise controlling, edge-preservation, and edge artifacts suppressing [14] can increase the SUVmax of a measured lesion, the other reason may be the different methods used for segmentation, as the automatic outline of MTV was based on the lesion’s SUVmax. BPL increased the SUVmean while decreased the MTV of tumor lesions, which results in no significant change in its TLG. Yamaguchi et al. confirmed that the BPL reconstruction algorithm is a feasible method for the suppression of edge artifacts deriving from PSF correction [14], because the edge suppression included in the BPL reconstruction allows suppression of excessive noise that would otherwise develop with high numbers of iterations. By limiting this noise, more iterations can be performed to enable full convergence of the algorithm. In our study, the maximum number of iterations in the BPL reconstruction as opposed to OSEM was 25, which can achieve full convergence of the algorithm. However, the suppression effect of BPL on noise is affected by sphere-to-background ratios and sphere size. When reconstructed by BPL, the suppression of edges is the most obvious and the boundary is sharpest when images of 10 mm spheres are at a high SBR and without background [14]. Additionally, our study further clarified that %ΔMTV was significantly positively correlated with the lesion size and the degree of the lesion’s uptake. However, due to the partial volume effect caused by the limited spatial resolution of conventional PET systems, radiotracer uptake is usually underestimated when lesions are three times smaller than the spatial resolution [15]. The partial-volume effect correction with a recovery coefficient method is important for improving the quantification accuracy of further clinical evaluations.

[^18^F]FDG-PET/CT quantitative parameters (such as SUVmax, SUVmean and MTV) as imaging metabolic indicators have unique advantages in clinical diagnosis, staging, post-treatment staging, and prognosis assessment of patients with malignant tumors [16–18]. The BPL reconstruction algorithm can improve the accuracy of quantitative parameters through effective convergence and promotes its utility as a biomarker of tumor metabolism. Meanwhile, accurate image segmentation is necessary for proper disease detection, diagnosis, treatment planning, and follow-up. Many PET-based automatic segmentation methods such as thresholding-based, stochastic and learning-based, region-based, boundary-based, and multi-modality methods have been proposed [19], but no consensus has been reached on the optimal delineation method. In this study, we chose the thresholding-based PET image segmentation method, which converts a gray-level image into a binary image by defining voxels greater than some value as the foreground and other voxels as the background [19]. The most frequent thresholding value used in the clinical setting is 40–43% of SUVmax, and we adopted a 42% thresholding value in the current study. Recently, a few more advanced PET image segmentation methods were studied, which are less sensitive to SUVmax variations such as: (1) a method that uses fully convolutional networks with auxiliary paths and uses dual-modality PET-CT images to achieve automatic segmentation of nasopharyngeal carcinoma on PET-CT images [20], which can achieve better performance than existing methods based only on CT images and purely fully convolutional networks; (2) a fully automatic and operator independent method based on an extension of the random walk algorithm for the BTV delineation of brain metastases for Gamma Knife treatments, which has the advantage of automatically identifying target and background random walk seeds and has an adaptive threshold to discriminate target from background voxels [21]; and (3) a new fully three-dimensional methodology for tumor delineation in functional images based on active contours and a slice marching approach, which are an active surface defined in the three-dimensional space and can segment all PET slices at once. The algorithm reduces the need for manual input to a minimum and produces tumor segmentations that are independent from the initial input, thus making the result extremely robust and repeatable [22]. However, these image segmentation methods are implemented on the basis of software application. As a clinician, it is difficult to make such a software application, and its clinical application is complex. Although the thresholding-based PET image segmentation method is vulnerable to SUVmax variations, it is easier to implement in clinical practice. Furthermore, based on this method, we obtained meaningful clinical results.

There are several limitations in the current analysis. Firstly, the number of lesions from different tumor entities is relatively small that the results obtained need to be verified in a larger cohort. Secondly, only data from clinical scans are presented and the ground truth for SUV and MTV is lacking, which leads to the uncertainty of diagnostic efficacy of these parameters. Thirdly, we only examined one set of reconstruction parameters for each of the algorithms although previous studies have shown that the differences between algorithms are dependent both on the settings for the OSEM-based algorithm as well as the beta parameter used with the BPL reconstruction [23]. Last but not the least, the thresholding-based PET image segmentation method used in our study is affected by SUVmax variations and may need considerable adjustments for different PET images.

## Conclusion

In conclusion, the BPL reconstruction algorithm significantly increases the SUVmax, SUVmean, and SBR of tumor lesions and decreases the MTV of tumor lesions compared with the OSEM and OSEM + TOF reconstruction algorithms. The lesion size and degree of lesion’s FDG uptake appear to be the main influencing factors in the different rates of PET/CT parameters between BPL and non-BPL reconstruction algorithms. The changes in small lesions and hypometabolic lesions are more obvious. Therefore, the advantage of BPL may help find out the small lesions with low degree [^18^F]FDG uptake and may facilitate more accurate diagnosis.

## Data Availability

The datasets generated and analyzed during the present study are available from the corresponding author on reasonable request.

## Abbreviations

BPL: Bayesian penalized-likelihood
PET/CT: Positron emission tomography/computed tomography
OSEM: Ordered subset expectation maximization
TOF: Time of flight
PSF: Point spread function
MTV: Metabolic tumor volume
TLG: Total lesion glycolysis
VOI: Volume of interest
SUV: Standardized uptake value
SBR: Signal-to-background ratio
